# Role of the Complement System in the Response to Orthopedic Biomaterials

**DOI:** 10.3390/ijms19113367

**Published:** 2018-10-27

**Authors:** Yvonne Mödinger, Graciosa Q. Teixeira, Cornelia Neidlinger-Wilke, Anita Ignatius

**Affiliations:** Institute of Orthopedic Research and Biomechanics, Centre for Trauma Research Ulm (ZTF Ulm), University of Ulm, D-89081 Ulm, Germany; yvonne.moedinger@uni-ulm.de (Y.M.), graciosa.teixeira@uni-ulm.de (G.Q.T.); cornelia.neidlinger-wilke@uni-ulm.de (C.N.-W.)

**Keywords:** complement activation, bone, inflammation, biomaterial, implant, orthopedics

## Abstract

Various synthetic biomaterials are used to replace lost or damaged bone tissue that, more or less successfully, osseointegrate into the bone environment. Almost all biomaterials used in orthopedic medicine activate the host-immune system to a certain degree. The complement system, which is a crucial arm of innate immunity, is rapidly activated by an implanted foreign material into the human body, and it is intensely studied regarding blood-contacting medical devices. In contrast, much less is known regarding the role of the complement system in response to implanted bone biomaterials. However, given the increasing knowledge of the complement regulation of bone homeostasis, regeneration, and inflammation, complement involvement in the immune response following biomaterial implantation into bone appears very likely. Moreover, bone cells can produce complement factors and are target cells of activated complement. Therefore, new bone formation or bone resorption around the implant area might be greatly influenced by the complement system. This review aims to summarize the current knowledge on biomaterial-mediated complement activation, with a focus on materials primarily used in orthopedic medicine. In addition, methods to modify the interactions between the complement system and bone biomaterials are discussed, which might favor osseointegration and improve the functionality of the device.

## 1. Introduction

Bone destruction induced by injury, infections, or bone diseases requires the replacement of the lost or damaged bone tissue by an adequate substitute. The use of autogenous bone grafts remains the gold standard, but allografts and demineralized bone matrices are also frequently implanted into bone defects. However, the limited availability of autogenous tissue and adverse immune responses towards allografts restrict their use and reveal the need for synthetic biomaterials [1].

A large number of different biomaterials are currently used, depending on the purpose of the implant device. For mechanically loaded regions, metals and metal oxides, that is, alumina and zirconia, are commonly used. To reconstruct bone defects, degradable ceramics and polymers have been developed, which can be applied, for example, as porous scaffolds, granules, injectable pastes, and gels, and loaded with cells, growth factors, and other biologically and pharmacologically active factors to guide bone regeneration or to prevent inflammation or infections at the implantation site [1].

The implantation process is normally accompanied by bone tissue trauma, in which the implant surface makes contact with blood, and is immediately covered with plasma proteins. Thereafter, successful integration into the surrounding bone and bone regeneration, in the absence of a fibrous capsule, are dependent on a balanced immune response towards the biomaterials. However, bone tissue can be markedly irritated by a foreign material. Persistent inflammatory reactions or infections can compromise bone formation and the function of a device, and even lead to implant failure [2]. Therefore, there is a need to better understand the immune responses at the implant–bone interface and how they influence osseointegration and bone regeneration.

There is increasing evidence that the complement system, a crucial arm of the innate immune system, plays an important role in bone homeostasis, regeneration, and inflammation [3,4]. Therefore, it is strongly anticipated that the complement system is also involved in the inflammatory processes towards bone biomaterials and might significantly shape bone–biomaterial interplay in the long-term. However, whereas the contribution of the complement system to inflammatory responses to blood-contacting implants, including artificial blood vessels and stents, has been studied extensively in recent decades [5,6,7], less is known about its role in the bone environment. The present review describes the current view on how the complement system influences the host response to foreign biomaterials used in orthopedic medicine. We aimed to summarize and discuss the role of complement within the immune reaction to a biomaterial and how it might affect implant osseointegration and bone tissue regeneration.

## 2. The Complement System and Its Activation by Artificial Surfaces

**Figure 1:**
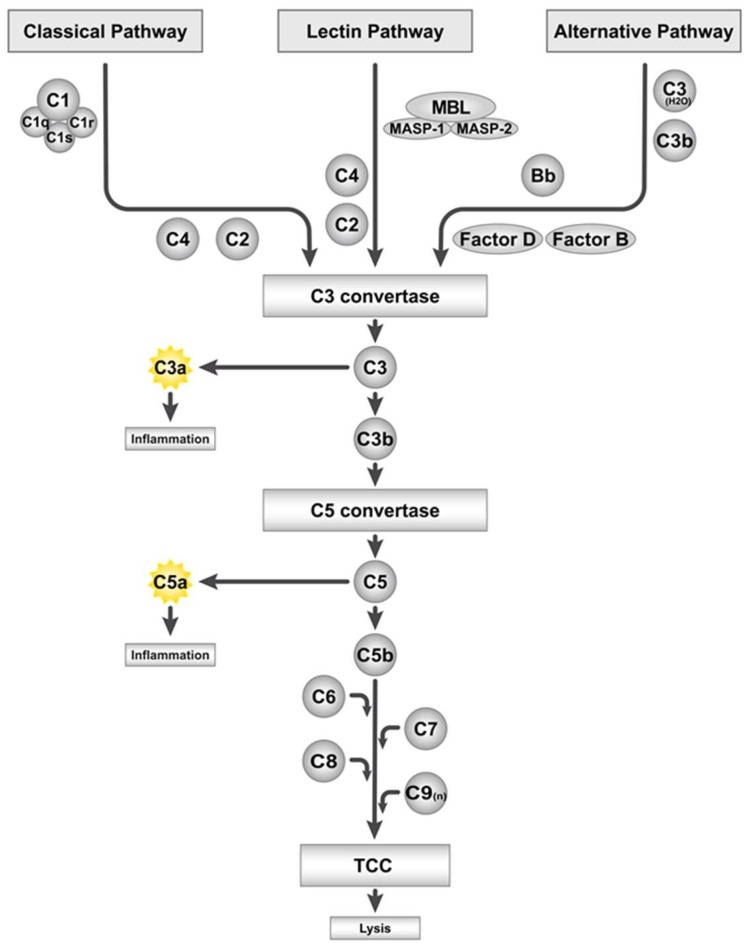
Overview of the complement system and its activation pathways. C: complement component, TCC: terminal complement complex, MBL: mannose-binding lectin, and MASP: MBL-associated serine protease.

The complement system is a humoral defense system of the innate immunity that recognizes danger signals evoked by intruding pathogens or body-intrinsic danger-associated molecular patterns (DAMPs) [8,9]. Activation of the complement system can occur via three different pathways, namely, the classical pathway, the alternative pathway, and the lectin pathway. Antigen–antibody immune complexes activate the classical pathway of the complement system. Hereby, complement component (C) 1 is activated by recognizing the antibody isotypes immunoglobulin (Ig) G or IgM of the immune complex, and the C1s subunit subsequently cleaves C4 and C2. The generated split products create a C3 convertase (C4bC2a) that cleaves C3 into C3a and C3b [10], as schematically depicted in Figure 1. The alternative pathway also leads to the formation of a functional C3 convertase (C3bBb), after low levels of C3 have been spontaneously hydrolyzed in a “tick-over” mechanism (C3(H2O)) and bind the factor B split product Bb, which is part of the alternative pathway C3 convertase. Initiation of the alternative pathway enables a rapid reaction to danger signals and potentiates the complement response in an amplification loop. The lectin pathway is induced when mannose-binding lectins (MBL) recognize carbohydrate sugar residues, which are present in a broad variety of bacterial cell membranes [10]. Similar to the classical pathway, this trigger leads to the cleavage of C4 and C2 and to the formation of a C3 convertase (C4bC2a), catalyzing C3 cleavage into its split products (Figure 1). While the anaphylatoxin C3a induces pro-inflammatory effects, including neutrophil and macrophage chemoattraction, the opsonin C3b binds to pathogens and induces their phagocytosis [9]. Moreover, C3b is part of the C3 and C5 convertases, the latter generating the anaphylatoxin C5a, which also acts as a pro-inflammatory and induces leukocyte attraction and activation via its receptors C5aR1 and C5aR2 (C5L2) [11,12]. The second split product C5b is a subunit of the terminal complement complex (TCC, C5b–9), a membrane pore-forming structure, which assembles on pathogenic surfaces to induce complement-mediated killing. In addition, the TCC can be present in its non-membrane-bound, soluble form (sC5b–9) [13,14].

Complement proteins are mainly generated in the liver, but other cells, including immune and bone cells, can also produce complement factors [15,16,17]. To ensure a balanced and restricted activation of complement, several complement regulatory proteins are described in the host, which have been reviewed recently [10,18]. Complement inhibition is a therapeutic approach to treat immune and inflammatory disorders, and drugs, including compstatin, a cyclic peptide that inhibits C3 cleavage; eculizumab, a C5-antibody; and C1-inhibitiors, have been developed, with the latter two being already in clinical use for treating paroxysmal nocturnal haemoglobinuria, atypical haemolytic uremic syndrome, and hereditary angioedema [19,20,21].

Biomaterials are known activators of complement [5,7,22], because they are foreign substances, and, in principal, every material alien to the body can activate the complement system. Complement activation is regarded as a crucial influence on the biocompatibility of artificial biomaterials. Surfaces and materials can be described as “complement activating” or “complement non-activating”. Intense complement activation was displayed by surfaces carrying hydroxyl groups [23,24,25] and free amino acids, which are hydrophobic rather than hydrophilic [7,23]. Furthermore, negatively charged materials with a large available surface have also been shown to activate the complement system [6]. The term ‘complement compatibility’, introduced by Tom Mollnes, describes the degree to which a biomaterial and foreign surface induces the complement system [26]. Notably, in contrast to the body’s native cells, which express complement regulators on their membranes to protect themselves from complement-mediated killing, artificial material surfaces lack such regulators, which favors uncontrolled complement activation.

Blood contact with a biomaterial immediately triggers the adsorption of large amounts of monolayered serum and plasma proteins into the surface. Upon surface binding, these proteins change their conformation and become “contact activated”. The newly exposed protein epitopes, which were previously masked, create a new interface between the material and the surrounding tissue that can strongly trigger complement activation [27,28,29]. C3 spontaneously binds to the initially formed protein layer and can ‘opsonize’ the material by completely covering its surface [30]. The split product C3b itself can also bind to the surface and form the alternative pathway convertase C3bBb. This further fuels the rapid amplification loop of C3 cleavage. The relative contribution of the three complement pathways to biomaterial-induced C3 cleavage remains to be fully determined. The main role has been assigned to the alternative pathway [5], a standard view that has been challenged because the classical pathway was also found to be involved, mainly in the early biomaterial recognition phase. Hereby, implant surface-adhered IgG recruits C1 and initiates the cascade of the classical complement pathway [31]. Therefore, both the alternative and classical pathways contribute to C3 convertase formation and C3b binding to biomaterial surfaces.

Surface-immobilized C3b activates and recruits leukocytes, because it is a ligand for complement receptor (CR) 1 (CD35) present on leukocytes [32]. In contrast, its inactive split product iC3b is bound by CR3 (CD11b/CD18), which is mainly expressed by monocytes, macrophages, and natural killer cells [33]. Furthermore, convertase-produced anaphylatoxins activate and recruit leukocytes to the implant site. Therefore, complement activation crucially influences immune-cell recruitment and interactions with the biomaterial, including their adhesion to the implant and the cytokine response [34].

Furthermore, complement was found to modulate the reaction to the implant by cross-talking with other biological systems, including the coagulation cascade and platelet activation [22,35].

Another aspect to be considered is the complement activation by infected implants and bacteria present at the implant site [2]. As the complement system per se is a defense system against intruding bacteria, understanding its role in biomaterial-related infection is crucial, particularly because complement activation is less controlled on non-self biomaterial surfaces, in contrast to host surfaces, given the lack of complement regulators. It is not entirely clear whether there is an increased infection risk when using complement-activating materials that can locally deplete complement, which is then unable to appropriately fight pathogens present at the implant site [36]. It was shown, for example, that biofilm formation on the implant reduced C3b deposition, thus allowing pathogen persistence through evasion from phagocytosis and neutrophil-mediated killing [37]. In contrast, it was proposed that bacterial defense might be improved by complement-activating surfaces, because the anaphylatoxins C3a and C5a are created at the implantation site [29].

In conclusion, biomaterial surfaces should be designed so that they limit both the response of complements to the surface itself, and pathogen-induced complement activation. Notably, orthopedic implants with various bactericidal and anti-adhesive surfaces are currently being developed or are already in clinical use, and modulation strategies include, for example, nanotopographical changes and surface-coating with ions, silver, or polymers [38].

The contribution of the complement system to inflammatory responses of blood-contacting materials has been studied intensely in recent decades [6,39]. In contrast, surprisingly little data are available on complement responses to biomaterials in bone. Here, it remains to be shown whether and to what extend complement activation is “the bad guy”, or whether it is the balanced activity between complement-activating, -regulating, and -inhibiting proteins in the peri-implant area that favors effective osseointegration.

## 3. Role of the Complement System in Bone

The complement system has been identified as a regulator of bone turnover, remodeling, inflammation, and repair in a number of studies [3,4]. This is unsurprising, because the immune and skeletal systems are closely interlinked, for example, through shared signaling molecules and because immune cells develop and mature in the bone marrow cavity. This concept of reciprocal regulation between bone and the immune system is termed osteoimmunology, and research therein has greatly increased in recent years [40,41].

Bone cells can locally produce complement proteins, including C3 and C5 expressed by osteoblasts [16,42,43] and active C5a generated by osteoclasts [17]. Notably, osteoblasts and their precursors, the mesenchymal stem cells (MSC), are chemoattracted by anaphylatoxins [44,45]. Moreover, C3a and C5a induce osteoblast release of inflammatory cytokines, including IL-6 and IL-8 [17,46,47], and C5a additionally stimulates the secretion of osteoclastogenic factors by osteoblasts [17,48]. Furthermore, it was shown that efficient osteoclast development requires the presence of the complement proteins C3 [49] and C5aR1 [50]. Together, these findings indicate that locally or systemically activated complement affects bone cell development, homeostasis, and cell-cell communication.

In addition to the described cellular effects, the importance of the complement system has been particularly recognized during bone inflammation and regeneration after bone injury [3,51]. Early inflammatory processes at the bone injury site involve the activation of the complement system, as shown, for example, by high C5aR1 levels at the fracture site of rats [44]. These data imply that C5a-C5aR1 signaling plays a crucial role in the response to bone fracture. Indeed, recent studies of our group confirmed this rationale, demonstrating that balanced C5aR1 activity is required during the entire course of bone fracture healing, because both the osteoblast-specific overexpression and a general knockout of C5aR1 resulted in disturbed bone healing [46,50]. Furthermore, we showed that not only C5a/C5aR-mediated actions affect bone regeneration, but also the TCC, which is formed downstream of the complement cascade. Herein, we demonstrated that reduced TCC levels not only disturbed fracture healing, but also resulted in a low bone mass phenotype, even under homeostatic conditions [52]. Related to this recent study, earlier findings showed that C5-deficienct mice, which have reduced C5b- and TCC-formation capacity, displayed diminished bone-healing capacity [53].

These diverse interactions between the complement system and bone might have direct implications for the osseointegration of biomaterials, which ideally occurs after the early inflammatory phase. The process of osseointegration can be described as having three distinct phases: osteoconduction, new bone formation, and bone remodeling [54]. Interestingly, this process is similar to the course of bone healing, which likewise consists of three subsequent but overlapping phases: inflammation, bone repair, and remodeling [55]. Given this similarity, an important role for the complement system in osseointegration appears likely and raises the question of how the immune environment and the complement system, in particular, affect bone cells around the implant site, and how this environment can be manipulated to favor osseointegration. The inflammatory response to the implant and its osseointegration might indeed be strongly affected, for example, by complement-mediated MSC and osteoblast migration, inflammatory osteoblast responses, and osteoclast formation.

Here, the open question remains whether excess complement activation either triggers peri-implant osteolysis, or contributes to an enhanced MSC and osteoblast recruitment to the implant site, and thus to a better functional integration of the foreign material.

## 4. Potential Role of the Complement System in the Immune Response to Bone Biomaterials 

The complement system is thought to contribute crucially to the foreign-body reaction towards orthopedic biomaterials, including, e.g., dental implants [56,57], which are characterized by the presence of large numbers of macrophages at the peri-implant area [58]. Macrophages recognize complement-opsonized implant wear particles via their complement receptors, leading to a constant trial and error of uptake by macrophages, the so-called “frustrated phagocytosis”. Macrophages then transform into multinucleated foreign body giant cells FBGC [59,60], which crucially define the foreign-body reaction to the implant. FBGC release oxygen free radicals, degrading enzymes, and cytokines, and contribute to osteolysis [56,61,62,63]. The accumulation of FBGC is considered a hallmark of orthopedic implant failure and peri-prosthetic osteolysis [61,64]. Nevertheless, some degree of foreign-body reaction towards the bone implant appears to be necessary for effective osseointegration [56,65] and is perceived as an inflammatory process modulated in concert with immune and bone cells.

**Figure 2:**
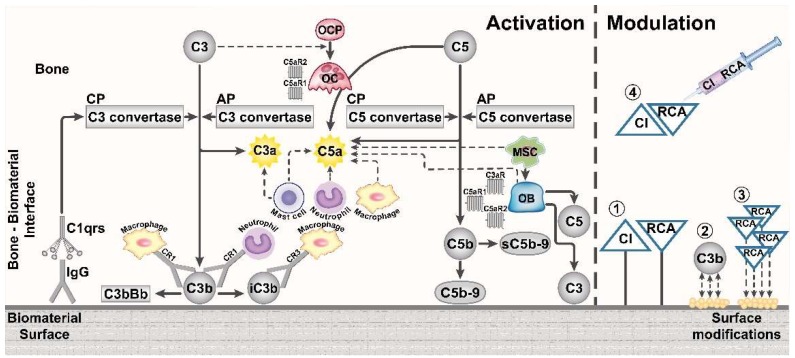
Complement activation by foreign biomaterial surfaces in bone tissue, and strategies to modulate the complement system. Complement activation and the presence of complement proteins (gray elements) in the bone-biomaterial interface and on the biomaterial surface are depicted. Both the classical pathway (CP) and alternative pathway (AP) convertases are regarded to contribute to complement activation in the response to biomaterials. In addition, the interaction with immune cells and their recruitment (dashed arrow line) to the implant site is illustrated. Furthermore, complement activation affects bone cells of the peri-implant bone tissue and their differentiation from precursor cells. Different strategies to modify complement activation in the bone implant area are depicted, including (1) implant coating with complement inhibitors (CI) and regulators of complement activity (RCA), (2) reduced complement activation by an altered adsorbed surface proteome to a modified surface, (3) implant coating with proteins that recruit RCA from the host, and (4) local application of CI and RCA. CR: Complement receptor, IgG: Immunoglobulin G, MSC: Mesenchymal stem cell, OB: Osteoblast, OCP: Osteoclast precursor cell, OC: Osteoclast, C3aR: C3a receptor, C5aR1: C5a receptor 1, C5aR2: C5a receptor 2 (C5L2).

The specific response of the complement system to different orthopedic biomaterials is yet to be investigated, to enable a tailored design of complement-modulating orthopedic surfaces. Detailed understanding remains lacking, for example, to what extent complement contributes to the early host reaction required for successful implant osseointegration and how excess complement activation can drive implant failure, whose manifestation can range from aseptic loosening and loss of structural support, to severe infection-related tissue degeneration around the implant. The following paragraphs and Figure 2 attempt to summarize the evidence for complement being “the good or the bad guy” in the response to distinct biomaterials used in bone tissue engineering.

### 4.1. Metals

Metals, including medical-grade stainless steel, cobalt-chromium alloys, titanium, and titanium alloys, have been used as load-bearing implant devices since the first attempts of orthopedic implant engineering and remain widely used [66,67]. Metals can also be applied as scaffolds or meshes in bone tissue engineering for load-bearing regions [68,69]. Even when metals, particularly titanium, are regarded as biocompatible and used with great success, problems can still arise due to corrosion and particle release [70,71,72].

An intact metal surface as well as metal particles can induce complement activation [73], which will be discussed in the following regarding titanium implants. In vitro, it was shown that titanium induces C3b, C3a, and C5a by C3 and C5 cleavage in human serum and plasma [74,75]. Titanium and its dioxide (TiO2) induce C3 cleavage by activation of the classical pathway and amplify this effect via the alternative pathway [76,77]. Titanium-surface modifications were found to play a pivotal role in controlling the degree of complement activation. It was shown, for example, that C3 preferably binds to smooth rather than rough titanium surfaces [78]. Furthermore, a rather simple surface treatment of titanium, by means of heat or ultraviolet (UV)-light, significantly decreased complement adsorption to the material [79]. Notably, UV-illumination of titanium was reported to improve early osseointegration in rat tibia [80]. To further address the effect of complement activation on titanium surfaces, in vivo bone healing and implant-anchorage were assessed upon implantation of titanium screws into rat tibiae, which were either complement-activating (IgG-coated) or non-activating (UV treated) [81]. The study revealed that the complement-activating surface induced greater inflammation and lower bone formation, but without a significant negative impact on bone anchorage, which was assessed as the end-point analysis after 4 weeks of healing [81].

Concluding, these studies imply a rather negative role for the activation of complement by titanium implants. Nonetheless, a recent study challenged this view, in which inflammation and osseointegration were assessed after titanium implantation in rabbit femurs [82]. As expected, titanium implantation evoked a foreign-body reaction. Yet, this activation appeared to promote osseointegration of the titanium implant, as reduced bone resorption and adequate new bone formation in the peri-implant area were observed. Moreover, the newly formed bone around the implant appeared more mature than that around the sham bone defect devoid of any material [82]. The authors correlated the titanium-induced osseointegration with complement, in that they detected significantly higher C5aR1 levels, but decreased C3 levels around the titanium implant. The reduced C3 levels were thought to suppress bone resorption [82], given the above-described osteoclastogenic action of C3 [49]. In addition, another recent investigation demonstrated that greater amounts of complement proteins adsorbed to surface-treated titanium after implantation. This altered adsorbed complement proteome was thought to increase implant bioactivity; however, bone repair was not significantly improved when implanting this surface-modified titanium, which got “complement-coated” in vivo, into rabbit tibiae [83].

Advances in in-depth proteome analysis by mass spectrometry could allow an unbiased characterization of the adsorbed proteome and “complementome” to in vivo implants. The presence of certain protein families could thereby be correlated to implant inflammation and the osseointegration process. Such experiments have been performed recently on serum proteins attached to silica–gelatin coated titanium implants in rabbit bone. The data imply that complement proteins, including C1, factor H, and C4b-binding protein, which were found to be enriched on the surface, crucially contributed to the fibrous capsule formation observed [84]. In contrast, the same group demonstrated that in case of an in vivo rabbit tibia implant, high levels of adsorbed complement proteins (C1q and factor D) to the coated titanium surface did not negatively affect osseointegration [85]. These studies show that detailed analysis of the implant-bound proteome and “complementome” might allow a prediction of the material’s behavior in vivo, which could be superior to current in vitro tests that do not sufficiently reflect the in vivo situation [86]. 

### 4.2. Ceramics

Medical ceramics include metal oxides, that is, alumina and zirconia, calcium compounds, like hydroxyapatite (HA) and tricalciumphosphate (TCP), and bioactive glasses [87]. Metal oxides are frequently used for load-bearing implants, including hip prostheses [88], whereas bioactive ceramics are applied as bone substitutes and in bone tissue engineering. Similar to metals, ceramics can activate complement, as shown for HA-coated dental implants that induce C3a and C5a generation in human serum [74]. In addition, calcium hydrogen phosphate, calcium carbonate, and TCP were found to induce C3 cleavage in vitro [89,90]. Moreover, complement deposition of C3 and C3b was found on zirconia, alumina, and HA surfaces [91,92], while C1s selectively bound to the latter two surfaces [92]. Interestingly, all investigated ceramic materials displayed a surprisingly low tendency to adsorb plasma proteins [92]. The structure of ceramics strongly influences complement activation, as demonstrated by Ferraz and co-workers. By increasing the nanopore size of alumina ceramics, greater amounts of C3 were adsorbed to the material, and increasing levels of C3a and sC5b–9 were detected in whole blood [93]. The herein described in vitro data on complement activation by ceramics remain to be linked to the possible in vivo behavior of ceramic implants.

### 4.3. Polymers

Several different synthetic polymers are currently used in orthopedic clinical practice or are proposed as promising future candidates. Among these are polyethylene (PE), polyglycolic acid (PGA), polystyrene, poly lactic acid (PLA), polymethylmethacrylate (PMMA), and poly-L-lysine (PLL). Moreover, various natural polymers, including collagen and chitosan, are increasingly considered as scaffolds for bone tissue engineering [94]. Studies investigating the complement activation potential of some of these polymers are summarized and discussed in the following paragraph.

PE is regarded as biocompatible, but the associated wear debris is considered to contribute to joint-implant failure by inducing osteolysis and aseptic loosening [95,96]. PE particles activate the alternative pathway of complement, demonstrated by the presence of factor Bb, C3a, iC3b, and sC5b–9 in synovial tissue specimens from revision patients with hip arthroplasty carrying a PE implant [95]. Additionally, polystyrene was shown to activate the alternative pathway by specifically interacting with factor D [97].

PMMA wear particles displayed C3d adsorption, and the blockade of CR3 specifically reduced PMMA phagocytosis by macrophages [98], suggesting that PMMA might be involved in peri-prosthetic osteolysis. In an earlier study, selected orthopedic materials and their particulates were tested in direct comparison for their C3 activation in vitro using human serum [99]. This study revealed that a high concentration of PE particles had the greatest ability to activate complement, whereas this effect appeared to be rather modest for PMMA and titanium [99].

The synthetic polymer PLL in the form of microspheres induced complement activation and cytokine production, while the addition of compstatin prevented these effects [100]. The same authors demonstrated that PLL microbeads significantly increased TCC levels in human whole blood, and that this could be reversed by the complement inhibitors compstatin and eculizumab [101].

PGA is a biodegradable and biocompatible material, used, for example, for fracture fixation [102]. In an experimental study, in which PGA pins were used intraarticularly for osteochondral fragment fixation, they induced swelling reactions and C5a generation [103], implicating a possible causal role of degraded PGA in complement activation. Confirming this, degraded PGA rather than PGA in its non-degraded form was found to strongly activate C3 deposition and anaphylatoxin production in vitro [104]. The mouse models used in this study, which were deficient in C1q, factor D, C2, factor B, or C6, demonstrated that degraded PGA activates both the classical and alternative pathway, and that inhibition of C5 may be a therapeutic option for limiting PGA-induced inflammation [104].

Polycarbonate, which is successfully used as a degradable scaffold in bone defects [105,106], did not display fluid-phase complement activation in serum [107]. This was in contrast to polyamide, which activated complement in the solid phase, thus on the material surface, and in the serum fluid phase [107].

Chitosan is a natural polymer, and as hybrid scaffold with silica it was shown to be suitable for bone-defect repair [108]. In a recent study, such a scaffold was assessed for the systemic presence of C3 after intramuscular implantation in mice [109]. C3 levels were increased in the early implantation phase, but not thereafter, and the scaffold displayed, overall, good biocompatibility and limited local inflammation [109]. Another study investigated complement activation by a non-absorbable scaffold made from saccharide agarose, which was seeded with autologous chondrocytes, in a canine model of cartilage-defect repair [110]. A strong foreign-body reaction towards the agarose scaffold, including fibrous capsule formation and high macrophage activity, induced complement activation and prevented growth and survival of the seeded chondrocytes. Other than intended, no newly formed cartilage was observed after scaffold implantation. The authors concluded that locally activated complement leads to death of the autologous cells [110]. These findings show that controlling complement activation against implanted scaffolds is important, particularly when colonizing the scaffold with living autologous or allogeneic cells to promote cartilage or bone repair.

Additionally, in the case of polymers, it was shown that the surface structure determines complement activation, which was decreased by decreasing the surface pore size, accomplished by nanostructured topography [6]. Moreover, differences in surface structure altered the recruitment of complement inhibitors, including C1-inhibitors [6]. This study shows that changing polymer surface nanotopography provides a simple means to adjust the tissue-implant-interface. Moreover, strong complement activation was observed by polymers formulated as hydrogels, whereas the same polymers did not evoke such responses when dissolved in solution [111]. Therefore, polymer state and conformation appear to be critical regarding the response of the complement system.

## 5. Modification of Biomaterial Surfaces to Influence Complement Activation

To reduce foreign-body reactions to orthopedic implants and to improve their osseointegration, physicochemical and biological modifications to biomaterial surfaces have been proposed [112,113]. Examples of such modifiable features are surface topography (e.g., pore size and roughness), stiffness, energy, and charge. Indeed, titanium bioactivity and osseointegration were significantly enhanced, for example, by surface alkali treatment, which increased the microroughness and hydrophilicity [114].

Future orthopedic biomaterials should be osteoimmunomodulative, thus having the ability to control both the local immune and bone environments to favor osteogenesis over osteoclastogenesis [115]. Therefore, modulating biomaterial-induced complement activation by various chemical, physical, and biological surface modifications might be a feasible approach to overcome adverse reactions towards the implant.

Indeed, the modification of surface properties strongly determines biocompatibility and complement compatibility [116]. For example, a reduction of complement activation by up to 50% was achieved when changing the surface nanotopography of a gold surface [117]. The altered surface structure is perceived to in turn alter the nature of the proteins adsorbed to the biomaterial. A recent review demonstrated that a customized surface structure is particularly important for orthopedic biomaterials, because it crucially shapes the implant-osseous tissue interface and modulates the effect of complement therein [112].

In addition to physicochemical modifications, current research also focuses on biological approaches to modifying complement responses, including surface coating with small bioactive molecules, which allows the shielding of biomaterials from complement activation [118]. Various methods of complement modulation and inhibition on biomaterial surfaces are described in detail by Ekdahl and colleagues [5]. In brief, two main biological approaches are pursued: (1) surface coatings, including with heparin, which evoke rather unspecific changes in complement activation, or (2) the linkage of defined complement regulators to the surface, which enables targeted inhibition and modification of complement proteins [119]. Heparin coating has been used efficiently for many decades as a solution for undesired complement activation on blood-contacting biomaterials [120,121,122]. Hereby, heparin is thought to bind complement regulators, including factor H, which is a complement regulator that displaces factor Bb from the alternative pathway convertase and mediates C3b decay. However, there are a broad variety of proteins that bind to heparin [123], and thus unwarranted effects could arise.

In contrast, the effectiveness of specific inhibitors to reduce complement activation by cardiovascular and cardiopulmonary bypass devices has been investigated. Among these complement regulators were soluble CR1; antibodies against factor D and C5; compstatin; C5aR-blockade; and surface-immobilized decay accelerating factor (DAF, CD55), a negative regulator of the C3 convertase [123,124,125]. Compstatin and a C5aR-blockade have also been proven to efficiently reduce complement activation in haemodialysis tubes and filters [126,127,128,129]. Furthermore, the direct linkage of factor H on model biomaterial surfaces, e.g., using polymer combinations, showed beneficial effects [130,131]. In another approach, surface coating with short peptides that will bind factor H was effective in reducing complement activation by a polystyrene surface [132]. This approach mimics, in principal, the immune evasion strategy of pathogens, which recruit complement regulators from the host to their cell membranes [133]. Moreover, bacteria themselves also produce complement-inhibiting factors to evade the killing mechanism of the host [3,134,135]. Importantly, polystyrene surface coating with such a complement-inhibiting bacterial peptide, derived from the M protein of Streptococcus pyogenes, was shown to greatly reduce the adverse effect against the biomaterial [136].

Taken together, the herein described complement-modifying approaches used for blood-contacting biomaterials could also be applied in orthopedic implants (Figure 2). However, differences in the implanted environment and in the material requirements between blood-contacting and orthopedic biomaterials should be considered. While complement inhibition appears to increase the haemocompatibility of blood-contacting devices, the same degree of inhibition might not be beneficial for the osseointegration of a bone biomaterial. Rather, a balance between complement activation and inhibition in the bone implant area might be an expedient solution, similarly as suggested in the case of bone repair following injury [3]. 

Imaginable surface modulation strategies comprise (1) surface coatings with complement regulators, (2) physicochemical surface modifications that alter the adsorption of complement proteins, (3) surface coatings with small peptides that bind complement regulators in situ, or (4) local or systemic short-term complement inhibition in the early implantation phase of the orthopedic device (Figure 2). Recent reviews summarized the current development stage of various complement inhibitors and regulators, which are discussed as promising therapeutics [19,21] and which could be considered in the design of complement-modulating orthopedic devices.

## 6. Conclusions and Perspective

Decreasing implant-related complications is of fundamental importance in orthopedics today, because patient morbidity and high costs for treatment and revision surgeries are associated therewith. Modulating the complement system and controlling its activation may also be a solution for orthopedic devices, in a similar way as described for materials being in direct contact with circulating blood [5,6,7]. 

To determine the feasibility of these approaches and to enable tailored design of surfaces that regulate specific complement components, it remains to be understood in detail how the complement system impacts the implant-surrounding bone tissue and how its modulation can shape osseointegration of the biomaterial. It remains unclear, for example, whether complement activation is an initial event or whether permanent-wear particle release of the implant activates complement in a constant manner. Furthermore, it is still unclear to what extent complement inhibition might be beneficial for osseointegration, and whether a blockade of the complement system might lead to a higher bacterial adhesion to the implant, thus increasing the risk for severe infections and implant failure.

However, most current studies and regulations regarding material-induced complement activation focus on in vitro assays using human serum or whole blood. These tests might be worthwhile regarding blood-contacting medical devices. However, for orthopedic implants, they will only reveal a limited part of the real in vivo situation, for example, by failing to consider complement-bone interactions. Furthermore, particles of bone implant materials used for in vitro testing frequently differ significantly in size and shape from those that arise in the clinical setting. Moreover, standardized in vivo models regarding the assessment of complement in osseointegration and bone repair following implantation are lacking. 

A constant and close collaboration between biomaterial engineers, medical researchers, and orthopedic surgeons is required in future studies to achieve complement compatibility of orthopedic devices and to improve the reliability of the test systems.
